# De novo assembly and annotation of the mangrove cricket genome

**DOI:** 10.1186/s13104-021-05798-z

**Published:** 2021-10-09

**Authors:** Aya Satoh, Miwako Takasu, Kentaro Yano, Yohey Terai

**Affiliations:** 1 Department of Evolutionary Studies of Biosystems, SOKENDAI (The Graduate University for Advanced Studies), Shonan Village, Hayama, Kanagawa 240-0193 Japan; 2 School of Agriculture, Meiji University, Kawasaki, Kanagawa 214-8571 Japan

**Keywords:** *Apteronemobius asahinai*, Circatidal rhythm, Illumina, Nanopore

## Abstract

**Objectives:**

The mangrove cricket, *Apteronemobius asahinai*, shows endogenous activity rhythms that synchronize with the tidal cycle (i.e., a free-running rhythm with a period of ~ 12.4 h [the circatidal rhythm]). Little is known about the molecular mechanisms underlying the circatidal rhythm. We present the draft genome of the mangrove cricket to facilitate future molecular studies of the molecular mechanisms behind this rhythm.

**Data description:**

The draft genome contains 151,060 scaffolds with a total length of 1.68 Gb (N50: 27 kb) and 92% BUSCO completeness. We obtained 28,831 predicted genes, of which 19,896 (69%) were successfully annotated using at least one of two databases (UniProtKB/SwissProt database and Pfam database).

## Objective

Some animals in the intertidal zone, which is influenced by a tidal flooding and ebbing cycle of approximately 12.4 h, show a tidal rhythm in their activity [1–3]. This endogenous rhythm, which persists even under constant conditions, is known as a circatidal rhythm, and it occurs over a range of ~ 11.5 h (predatory mite) [4] to ~ 13.8 h (high-shore limpet) [5]. Although the molecular mechanisms underlying the circadian rhythm (i.e., an endogenous rhythm with a period of ~ 24 h) are well known [6], mechanistic studies of circatidal rhythms are limited [7, 8].

The mangrove cricket (*Apteronemobius asahinai*), an endemic species of mangrove forest floors, is also influenced by tides. This cricket shows a circatidal rhythm in its locomotor activity, with a period of ~ 12.6 h [9, 10]. This endogenous rhythm is not entrained by the light–dark cycle but by periodic inundations [11, 12]. The mangrove cricket is one of only a few model organisms studied for the purpose of understanding the molecular mechanisms of the circatidal rhythm. Previous work demonstrated that the circatidal rhythm was not disrupted by suppressing the expression of two circadian clock genes, *period* and *Clock* [13, 14]. These findings indicate that the molecular components of the circatidal clock differ from those of the circadian clock in the mangrove cricket. Recently, transcriptome analyses of this species were conducted to reveal circatidal clock-controlled genes [15] or to identify biological processes related to the circatidal rhythm [16]. Here, we provide the draft genome of the mangrove cricket. This information is expected to contribute to future molecular studies by enabling the use of molecular techniques such as GWAS.

## Data description

Mangrove crickets were collected from a mangrove forest in Ginoza, Okinawa Prefecture, Japan. To generate highly homozygous individuals, we repeated sibling mating over 7 generations and used two adult males of the eighth generation for DNA extraction (for details, see Data file 1). Genomic DNA from the whole body of a male was extracted using the DNeasy® Blood & Tissue Kit (Qiagen). The NEBNext Ultra II DNA Library Prep Kit for Illumina (New England BioLabs) was used to construct a library from 500 ng sample DNA. Paired-end (2 × 150 bp) sequencing was performed on the Illumina HiSeq X platform. For long-read library preparation, genomic DNA from the whole body of another male was extracted using the DNeasy® Blood & Tissue Kit and Genomic-tip 20G Kit (both from Qiagen). Short DNA fragments were removed using Short Read Eliminator Kit (Circulomics). The library was constructed from 415 ng sample DNA using the Rapid Sequencing Kit (SQK-RAD004; Oxford Nanopore Technologies [ONT]). Sequencing was performed twice on the MinION Mk1b with a flow cell R9.4 (FLO-MIN106D; ONT). The Illumina and ONT platforms yielded 217.5 and 14.6 Gb of nucleotide sequence, respectively. The Illumina reads (Data file 2) were assembled and scaffolded using the CLC genomic workbench v20.0.4 [17]. The ONT reads (Data file 3) were trimmed for adapter and low-quality reads using Porechop v0.2.4 [18] and Nanofilt v2.8.0 [19], respectively, and then error-corrected using the Illumina reads by LoRDEC v0.9 [20]. Finally, the error-corrected ONT reads were subjected to gap closing in the scaffolds using TGS-Gapcloser v1.1.1 [21]. The final draft genome (Data file 4) consists of 151,060 scaffolds with a total length of 1,676,217,857 bp, average length of 11,096 bp, and N50 of 27,317 bp. BUSCO analysis using the online interface gVolante [22] identified 983 genes (92.21%) among the 1,066 arthropodal universal orthologs completely, and only 17 genes (1.59%) were missing, indicating high completeness of our draft genome.

RepeatModeler v2.0.1 [23] estimated 2532 repeat sequences, which were utilized by RepeatMasker v4.0.9 [24] to mask the repetitive elements in the genome. The repeat sequences in the assembly comprised 572,734,587 bp (34.17% of the total length). The MAKER v2.31.11 [25] pipeline predicted 28,831 protein-coding genes in the hard-masked genome (Data files 5–7). The average coding sequence length was 997.08 bp, with an average intron length of 1000.45 bp and average number of exons per gene of 4.34. We annotated 16,528 genes (57.3%) via a BLASTP v2.10.1 + [26] search (E-value threshold of 1 × 10^–10^) against known proteins in the UniProtKB/SwissProt Database [27]. InterProScan v5.50–84.0 [28] identified 4537 domain families among 17,932 (62.3%) genes via a search of the Pfam database. As a result, 69% of the predicted genes were successfully annotated by at least one of the two methods.

## Limitations

The genome size, assessed by the k-mer frequency distribution of the Illumina reads using KmerGenie v1.7051 [29], was estimated to be 1,610,998,267 bp. Based on this estimation, the sequencing depths obtained from the Illumina and ONT platforms were calculated to be 134× and 9× , respectively. Since the coverage of ONT reads was low, the usage of them were limited only to the gap closing. The genome size of the mangrove cricket is comparable with the three previously sequenced Gryllidae genomes: *Teleogryllus occipitalis* (1.93 Gb) [30], *Teleogryllus oceanicus* (2.05 Gb) [31], and *Laupala kohalensis* (1.6 Gb) [32].

## Data Availability

The data described in this Data note can be freely and openly accessed on DDBJ under BioProject ID: PRJDB11838 and the figshare database. Sequence reads have been deposited at DDBJ Sequence Read Archive under accession number DRX290103 (https://identifiers.org/insdc.sra:DRX290103) [34] and DRX290104 (https://identifiers.org/insdc.sra:DRX290104) [35]. The whole genome sequence data has been deposited at DDBJ under accession number BPSV01000000 (https://identifiers.org/ncbi/insdc:BPSV01000000) [36]. The other data files generated in the current study are available at the figshare database: Data file 1 (https://doi.org/10.6084/m9.figshare.16632781) [33], Data file 5–7 (https://doi.org/10.6084/m9.figshare.14746056) [37–39]. See Table 1 and references [33–39] for details.

**Table 1:** Overview of data files/data sets

Label	Name of data file/data set	File types (file extension)	Data repository and identifier (DOI or accession number)
Data file 1	Materials and Methods	PDF	figshare: https://doi.org/10.6084/m9.figshare.16632781 [33]
Data file 2	Illumina raw sequences	FASTQ	DDBJ Sequence Read Archive: https://identifiers.org/insdc.sra:DRX290103 [34]
Data file 3	ONT raw sequences	FASTQ	DDBJ Sequence Read Archive: https://identifiers.org/insdc.sra:DRX290104 [35]
Data file 4	Whole genome sequence data	FASTA	DDBJ: https://identifiers.org/ncbi/insdc:BPSV01000000 [36]
Data file 5	Structural and functional gene annotation	GFF	figshare: https://doi.org/10.6084/m9.figshare.14746056 [37]
Data file 6	predicted protein sequences	FASTA	figshare: https://doi.org/10.6084/m9.figshare.14746056 [38]
Data file 7	predicted transcript sequences	FASTA	figshare: https://doi.org/10.6084/m9.figshare.14746056 [39] Overview of data files/data sets

## Abbreviations

BUSCO: Benchmarking Universal Single-Copy Orthologs
Gb: Giga base pair
GWAS: Genome-wide association studies
kb: Kilo base pair
ONT: Oxford Nanopore Technologies
